# Debate on the Morphological Variability of the Lateral Pterygoid Muscle—Discrepancies, Speculations and New Original Anatomical Samples

**DOI:** 10.3390/medicina60121913

**Published:** 2024-11-21

**Authors:** Mugurel Constantin Rusu, Corneliu Toader, Răzvan Costin Tudose, Laura Octavia Grigoriţă

**Affiliations:** 1 Division of Anatomy, Faculty of Dentistry, “Carol Davila” University of Medicine and Pharmacy, 020021 Bucharest, Romania; razvan-costin.tudose0721@stud.umfcd.ro; 2 Division of Neurosurgery, Department 6–Clinical Neurosciences, Faculty of Medicine, “Carol Davila” University of Medicine and Pharmacy, 020021 Bucharest, Romania; corneliu.toader@umfcd.ro; 3 Clinic of Neurosurgery, “Dr. Bagdasar-Arseni” Emergency Clinical Hospital, 041915 Bucharest, Romania; 4 Department of Anatomy, Faculty of Medicine, “Victor Babeş” University of Medicine and Pharmacy, 300041 Timişoara, Romania; grigorita.laura@umft.ro

**Keywords:** masticatory muscles, lateral pterygoid muscle, temporomandibular joint, mandible

## Abstract

The lateral pterygoid muscle (LPM), a critical component of the masticatory muscles, typically comprises upper (SLPM) and lower (ILPM) heads. However, it is essential to note that the LPM’s structure is not a constant feature, as the number of bundles and their topography can vary. Moreover, additional heads, such as medial and middle heads, and unique-headed configurations of the LPM have been reported. Several studies have demonstrated the penniform structure of the LPM, which is further supported by its diverse pattern of innervation. Anatomically, the LPM originates from the greater wing and lateral pterygoid plate of the sphenoid bone, with variations in these origins being common. For instance, the presence of a broad lateral pterygoid plate or extensions from it can enlarge the origin area of the LPM. Equally variable are the insertions of the LPM, which can include attachments to the mandibular condyle and the temporomandibular joint disc. In some cases, aberrant LPM bundles may attach to the mandibular condyle outside the mandibular notch. Rarely encountered muscles like the pterygoideus proprius, pterygospinosus, and pterygofacialis further add to the diversity of this muscle. The anatomy of the LPM is subject to modification due to factors like atrophy or hypertrophy. Therefore, it is imperative to recognize that a one-size-fits-all anatomical pattern for the LPM does not exist. Instead, a personalized therapeutic approach should be based on a case-by-case determination of the LPM’s specific anatomical configuration. This nuanced understanding challenges the simplistic view of the LPM and underscores the need for individualized clinical considerations.

## 1. Introduction

In the head, there are two main groups of muscles: muscles related to the facial expression and masticatory muscles [1,2]. These latter muscles are on each side: the temporal, masseter, lateral, and medial pterygoid muscles. The lateral pterygoid muscle (LPM) is anatomically the most versatile. The human LPM plays an essential role in the control of jaw movements [3]. The morphology of the LPM is still under debate because of its deep location and the difficulties in approaching it for study [4,5]. The different preparation techniques seem to be the main reason for the diverging results [4].

The LPM is responsible for three main mandibular movements: laterality, by unilateral contraction; protrusion, by simultaneous bilateral contraction; and full mouth opening when its bilateral contraction follows the action of the hyoid muscles [6]. In the temporomandibular joint (TMJ), the LPM is the only muscle that attaches directly to the condyle and the articular disc and has no direct antagonistic muscle insertion on the opposite posterior sites [7]. It is nearly impossible to palpate the LPM anatomically [8].

Controversial opinions regarding the origin and insertion of the LPM make it challenging to understand its function [9]. Despite its clinical significance, the anatomy of the human TMJ and its relationship to the LPM remain poorly described and often misrepresented in standard texts [9]. The LPM is mainly involved in temporomandibular disorders, a common form of non-dental chronic orofacial pain [10]. Studies on the anatomy and function of the LPM have influenced dental clinical practice [11]. However, there are almost as many anatomical and physiological assumptions about the LPM as there are publications [12]. A systematic review of the morphological variations of the LPM studied by different methods was documented by Stockle et al. (2019), who included 81 papers in that study [4]. Of these, 69 used anatomical methods of study, 5 used imaging methods and 7 studies used combinations of the two methods [4]. We adhere to these authors’ comment regarding the fact that magnetic resonance tomography is an adequate method to explore the morphology of the LPM, because the anatomic proportions, the pathological alterations and the surrounding soft tissues can be well depicted without any harmful invasive intervention [4]. We, therefore, sampled this debate with original pictures from cases we used in previous studies [13].

The LPM is known for its anatomical variability, particularly in terms of the number of heads. The classical anatomical literature commonly describes the LPM as having two heads: a superior (sphenoid, SLPM) and an inferior (pterygoid, ILPM) head [14,15,16,17]. These two heads are believed to be distinct anteriorly but to merge posteriorly near the TMJ. The two-headed structure is widely accepted, with several studies reporting a 100% prevalence of this variant [18,19]. Nevertheless, in the approved Anatomical Terminology of 2019 (TA2) are listed the superior head of the LPM (caput superius musculi pterygoidei lateralis) and the inferior head of the LPM (caput inferius musculi pterygoidei lateralis) [20]. The SLPM is regarded as the sphenomeniscus muscle [20].

We, therefore, aimed to document the multiple variables that determine the morphological and functional heterogeneity of the LPM and the available information about it. The literature search for this study was conducted using PubMed, Web of Science, Scopus, and Google Scholar, focusing exclusively on English-language papers published up to December 2023. We accessed all the identified articles in full-text form, ensuring a comprehensive analysis of the available literature. Quality assessment of the included studies was performed using the Newcastle-Ottawa Scale (NOS). This study encompasses systematic reviews and original research to provide a thorough perspective. Data extraction and quality evaluation were collaboratively executed by the authors, with discrepancies resolved through joint reanalysis and consensus. This methodological approach ensures the credibility and relevance of our opinion. Original findings were also used to support rare or unknown anatomical variations associated with the LPM.

We discuss here how the LPM fits with typical anatomical descriptions, structural studies of the LPM, modified attachments of this muscle, and volume modifications of it.

## 2. Previous Studies on the Lateral Pterygoid Muscle

Different methods were used previously to study the LPM, dissections [5,9,16,17,21,22,23,24,25,26,27,28,29,30,31,32,33,34,35,36,37] or MRI [19,38,39,40,41,42]. Numerous studies used less than 30 specimens to approach the LPM [9,16,17,21,22,23,25,26,27,30,31,33,34,35,36,37,43,44].

Sugisaki et al. (1986) observed that the LPM was substantially different from cadaver to cadaver and that the muscle fibers were so crowded with each other and with those of the temporal muscle that only a few cases were found with the SLPM and ILPM completely separated from each other [21]. Widmalm et al. (1987) observed that at the origins of the LPM heads are regions in which the fibers are interlaced or closely overlapped by fibers of either the temporal or the medial pterygoid muscle [22]. According to the positions of the origins and insertions of the LPM and the positional relationships to the nerves, the muscle was not clearly divided into heads in a study by dissection of 94 sides [32]. Heterogeneous results regarding the LPM insertions also resulted from these studies [16,19,21,22,23,25,26,28,29,30,31,35,36,37,39,41,43].

## 3. The Anatomy of the Lateral Pterygoid Muscle

### 3.1. Origin of the Lateral Pterygoid Muscle

**Figure 1:**
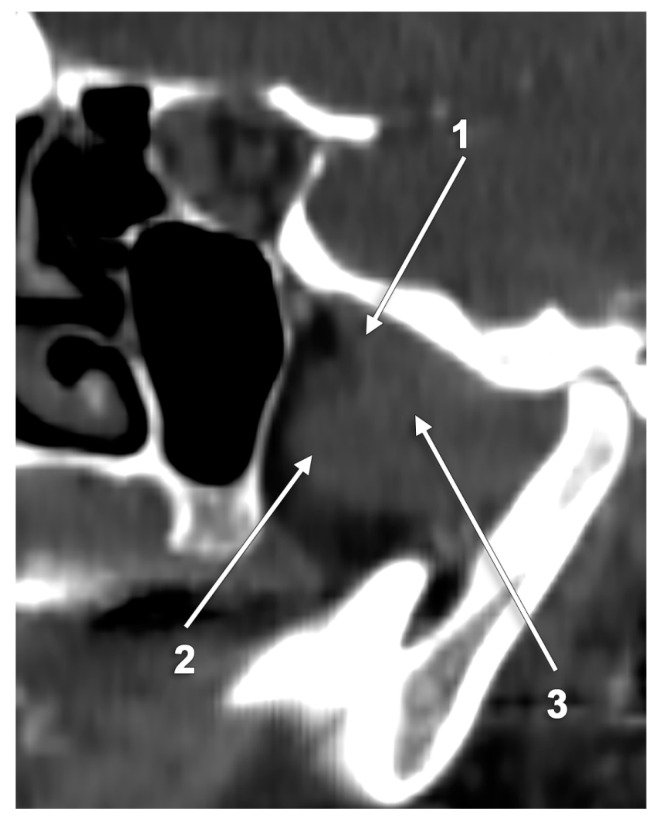
Sagittal CT slice through the lateral pterygoid muscle: 1. superior head; 2. inferior head; and 3. merged heads.

According to *Gray’s Anatomy*, the LPM consists of two parts with different origins: the superior and inferior heads [2]. However, indicating the “parts” of the LPM suggests the muscle is divided, whereas indicating the “heads” implies that these should merge posteriorly. Different authors used “heads” while discussing the LPM, but they depicted “parts” in their illustrations or figures [19,35,37,39,41,42]. Other authors indicated the two parts of the LPM as “bellies” [5,8], neither well defined [5]. The LPM parts were indicated as “heads” and “lobes” without discrimination [42]. Rouviére and Delmas described instead two bundles of the LPM with different origins, a small superior (SLPM) or sphenoidal bundle and, respectively, a large inferior (ILPM) or pterygoid bundle [1]. The two bundles merge before being inserted into the TMJ disk and the neck of the mandible [1] (Figure 1). The superior fibers are flattened vertically and obliquely postero-laterally, but the inferior fibers are flattened in the transverse plane [12].

The two heads of the LPM were shown in humans to have different, if not antagonistic, functions [45]. To avoid confusion, it was suggested that the SLPM might be called the superior pterygoid muscle [45]. Christensen (1969), as quoted by McNamara (1973), observed that the two heads of the LPM in humans are distinct anatomical entities [46].

The SLPM originates from the infratemporal face, infratemporal crest, and sphenoidal tubercle (infratemporal spine) of the greater sphenoidal wing [2,47]. The outer side of the non-lamellar pterygoid root was also included in the origin sites of the SLPM [12].

The bony landmarks for the ILPM origin were listed as follows: the outer side of the lateral pterygoid plate, the maxillary tuberosity, and the lateral surface of the pyramidal process of the palatine bone [12,16]. The tuberal head is consistently present, but the origin on the pyramidal process of LPM fibers is occasional [47]. *Gray’s Anatomy* did not indicate the ILPM origins on the pyramidal process and maxillary tuberosity [2]. Lang described that the ILPM arises from the lateral surface of the pterygoid plate and from “a narrow strip on its maxillary surface” [47]. The upper fibers of the ILPM run more horizontally, and the lower fibers are more oblique [47].

**Figure 2:**
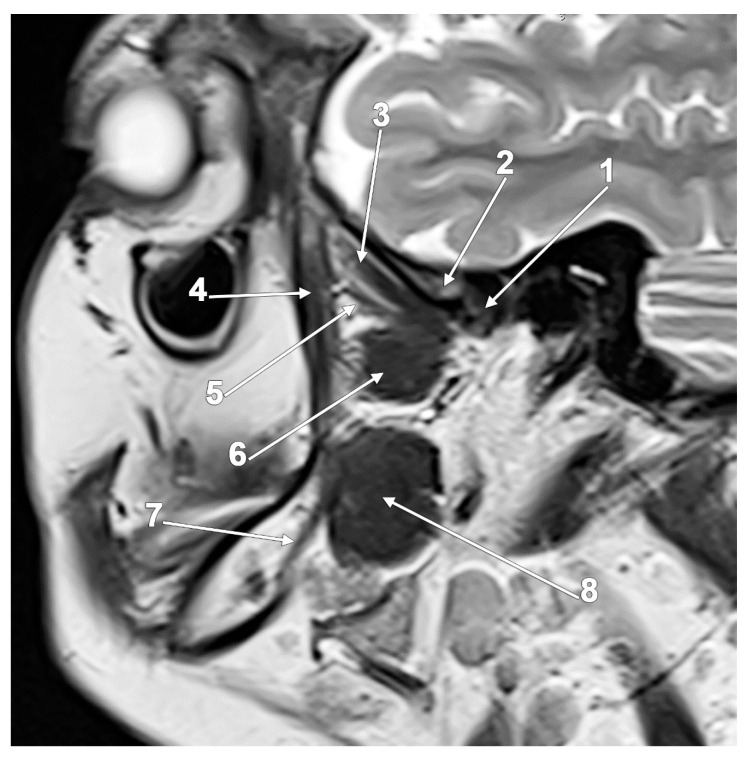
Sagittal MRI slice through the lateral pterygoid muscle (LPM), where three heads/bundles are found: 1. head of the mandible; 2. articular tubercle; 3. superior head of the LPM; 4. deep fibers of the temporal muscle; 5. middle head of the LPM; 6. inferior head of the LPM; 7. mandibular canal; and 8. medial pterygoid muscle.

At its origin, the LPM may have two or three heads [48], but this does not necessarily mean they do not merge toward the insertion sites (Figure 2). When a middle head (MLPM) is found between the superior and inferior ones, it originates on the lateral pterygoid plate above an inferior head, which is narrower than usual [49]. The typical anatomy of the LPM was used in biomechanical studies. A linear programming model analyzed the contributions of the LPM to a variety of different tasks. The study used just a two-headed model of the LPM: the SLPM and the ILPM [50].

### 3.2. Insertions of the Lateral Pterygoid Muscle

**Figure 3:**
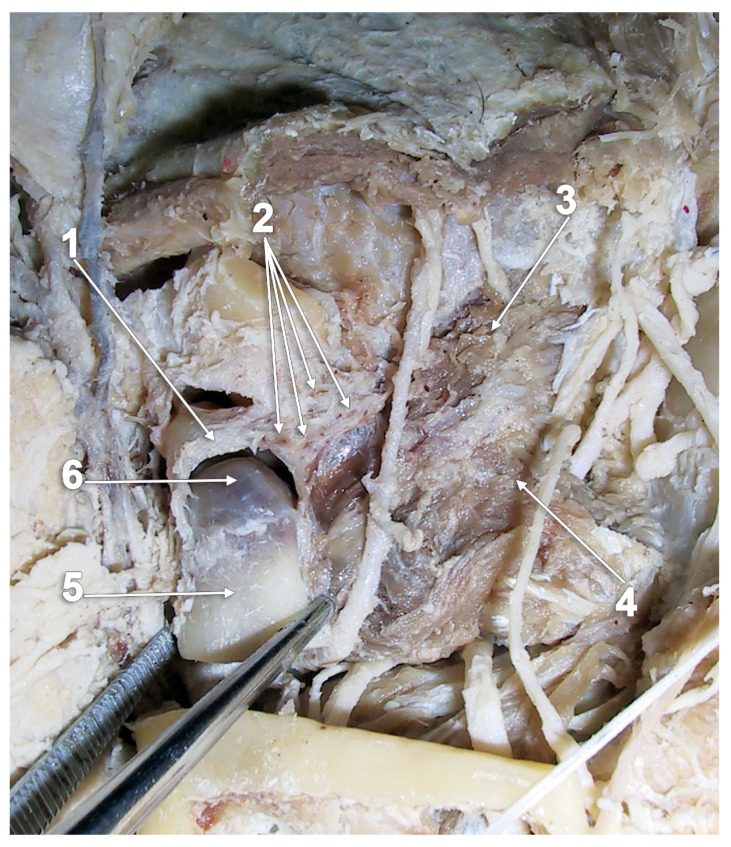
Dissection of the right temporomandibular joint (TMJ) and lateral pterygoid muscle (LPM). Lateral view: 1. TMJ disc; 2. muscle fibers intermingled with the articular capsule and anterior disc extension; 3. superior head of the LPM; 4. inferior head of the LPM; 5. neck of the mandible; and 6. head of the mandible.

**Figure 4:**
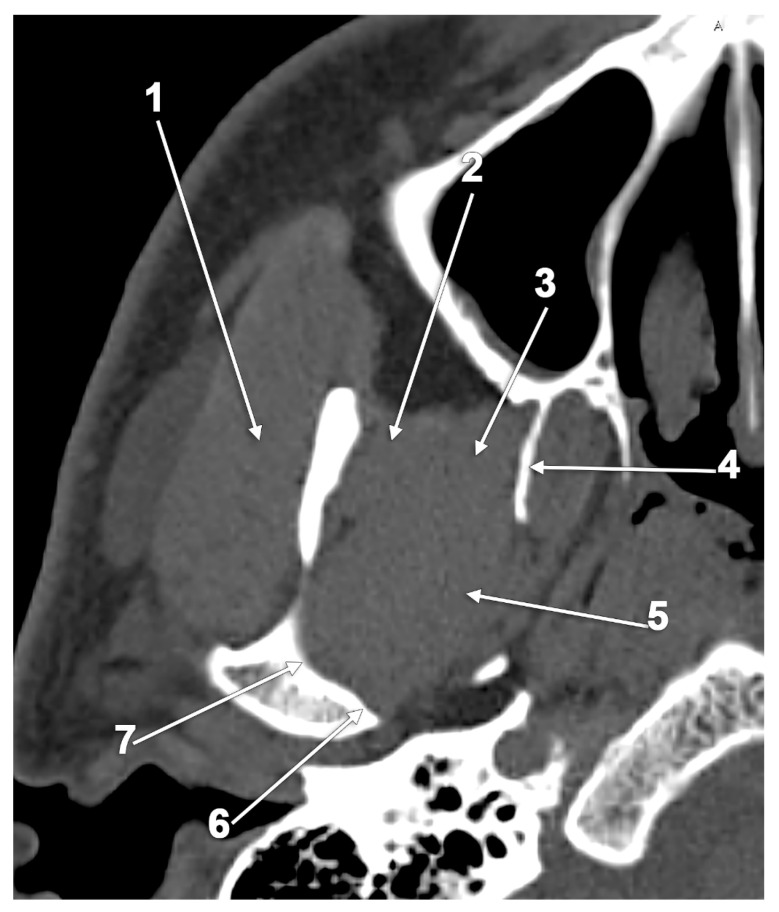
Axial CT slice through the inferior head of the lateral pterygoid muscle (ILPM): 1. masseter m.; 2. temporal m.; 3. ILPM fibers from the anterior half of the lateral pterygoid plate; 4. lateral pterygoid plate; 5. ILPM fibers from the posterior half of the lateral pterygoid plate; 6. medial condylar fovea; and 7. pterygoid fovea.

According to *Gray’s Anatomy*, the LPM’s merged heads insert onto a depression on the front of the mandible’s neck (i.e., the pterygoid fovea), and a part of the upper fibers may be attached to the capsule of the TMJ and the anterior and medial borders of the TMJ disc (TMJD) [2]. These could be observed on dissected cadavers (Figure 3). The LPM insertions are typically internal to the mandibular notch. However, the percentage of fibers of the SLPM inserted into the TMJD is debated in the literature [18]. The insertion of the SLPM into the TMJD ranges from a fleshy mass to a few fibers [51]. Different studies suggest that the SLPM can be defined as “sphenomeniscus”, “protrudens menisci” or “pterygoideus” [9]. However, all the SLPM fibers gain direct or indirect insertion into the condyle of the mandible [23]. The ILPM fibers from the anterior half of the lateral pterygoid plate insert into the pterygoid fovea and the fibers from the posterior half of the lateral pterygoid plate mainly insert into the medial surface of the condylar process, on a medial condylar impression or fovea immediately below the medial condylar pole [32,36] (Figure 4). The attachment of the fibers along the medial surface of the mandibular condyle (MC) and TMJD helps to maintain the stability of the joint and to stabilize the condyles during unilateral forward protrusion [52]. In addition, these medial fibers are a factor in guiding the bilateral forward protrusion of the mandible, as well as its bilateral retrusion, which is of great importance to prosthodontists in establishing the centric relation [52].

### 3.3. Innervation of the Lateral Pterygoid Muscle

It is generally described that the LPM is innervated by the buccal nerve [32]. Akita et al. (2000) found that twigs of the anterior deep temporal nerve were distributed to both the SLPM and ILPM, twigs of the middle deep temporal nerve were distributed to the SLPM, and twigs of the mandibular nerve trunk to the ILPM [53]. Twigs from the auriculotemporal nerve were found innervating the ILPM from the medial aspect near the insertion [54]. A masseteric nerve supply of the LPM was not found during an LPM study by dissection [54], although it was previously reported [55]. The patterns of innervation of the LPM were studied for the superior part of the SLPM, the inferior part of the SLPM, and the ILPM by Davies et al. (2012) [17]. There were found to be independent sources of innervation to each of the quadrants in the superior part of the SLPM (masseteric/posterior deep temporal/middle deep temporal/buccal), but one primary source of innervation (buccal) to the quadrants of the inferior part of the SLPM [17]. The buccal nerve supplied both the medial and lateral quadrants of the ILPM, with the medial quadrants receiving additional innervation from muscular branches of the mandibular nerve [17]. Previously, Kim et al. (2003) found that the buccal nerve supplied the SPLM in just 45.8% of cases and the nerves innervating the ILPM originated from both the buccal and mandibular nerves in 58.3% of cases [27]. The buccal nerve innervated both the SLPM and ILPM in just 20.8% of cases, while in 45.9% of cases, additional muscular branches of the mandibular nerve were distributed to the ILPM [27]. Foucart et al. (1998) discussed that according to its nerve supply, the LPM must be considered a single unit made of independent functional musculoaponeurotic layers even though its morphologic conformation is in a variable number of heads [55].

#### The Reciprocal Innervation of the Lateral Pterygoid Muscle Heads

The classical description of the normal function of the LPM is that the SLPM is active in jaw-closing, jaw retrusion, and ipsilateral jaw movements, while the ILPM is active in jaw-opening, jaw protrusion, and contralateral jaw movements [3]. Electromyographic data from verified recording sites within the SLPM indicate, however, that it plays a role in contralateral, protrusive, and jaw-opening movements similar to the ILPM [3].

Electromyographic studies have demonstrated that the SLPM and ILPM are reciprocally innervated so that the ILPM contracts during mouth opening while the SLPM relaxes, and the situation reverses during mouth closure [2]. A functional survey in Macaca Mulatta found that the SLPM contracts during the elevation of the mandible and, variably, during swallowing, and the ILPM is active during the opening of the mouth [56]. Osborn (1995), as quoted in [2], offered an explanation for this surprising activity as follows [57]. Most of a clenching force’s power is due to masseter and temporalis contractions. Still, the associated backward pull of the temporalis is greater than the forward pull of the masseter, so their combined jaw-closing action potentially pulls the MC backward [57]. This is prevented by the simultaneous contraction of the SLPM, which stabilizes the condylar head against the articular tubercle during mouth closure, particularly during biting and chewing [57]. This view, however, is based on studies of electromyographic activity from the LPM, where the electrode recording sites were not verified by some form of imaging to avoid inadvertent recordings from other masticatory muscles [3].

Murray et al. (2004) performed a series of experimental studies on the LPM [11]. Based on their results, these authors agreed with a previous theory documented by Hannam and McMillan (1994) that both heads of the LPM constitute “a system of fibers acting as one muscle, with varying amounts of evenly graded activity throughout its entire range, with the distribution ‘shaded’ according to the biomechanical demands of the task” [11,58]. This theory was initially supported by an electromyographic study by Widmalm et al. (1987) in just five subjects [22]. Hannam and McMillan (1994) considered in their review of the LPM that the muscle “is partitioned anatomically in a straightforward fashion and seems to be designed to function differentially more by its separate origins than by any complex internal arrangement of septa” [58]. The results of Murray et al. (2004) suggested that the LPM is functionally analogous to the fan-shaped temporalis muscle, which has a continuous range of functional properties from posterior to anterior [11]. They believed that the LPM “has a continuous range of properties from superior to inferior and from medial to lateral” and “the concept of a specific pattern of coordination between superior and inferior heads has little meaning” [11]. This is because motor units within the LPM will be recruited as determined by the biomechanical demands of the task [11].

Later, Murray (2012) suggested that both the SLPM and ILPM are functionally heterogeneous; different parts of each head of the muscle can be activated independently of other parts [3]. The results of electromyographic studies of the LPM referred to a typical structure with two heads. Seemingly, the functional heterogeneity of the LPM could be correlated with a heterogeneous morphology that was not or could not have been tested during the electrodes’ insertion.

### 3.4. Vascularization of the Lateral Pterygoid Muscle

The LPM receives direct muscular branches of the maxillary artery [59]. An arterial supply of the LPM from the ascending palatine artery was also indicated [47].

## 4. The Structure of the Lateral Pterygoid Muscle

The LPM is the only masticatory muscle with horizontally arranged fibers [4]. However, different structural patterns of the LPM have been reported or reviewed [60].

The internal architecture of the LPM, despite its apparent variations, tends to exhibit a more straightforward structure than one might anticipate [58]. It comprises relatively long fibers, measuring about 22 mm in length [61], a notable characteristic differentiating it from other masticatory muscles [58]. These fibers, akin to the posterior fibers of the temporalis muscle, align along the muscle’s line of action. This particular alignment is more conducive to elongation than to generating significant power, contrasting with muscles like the masseter and medial pterygoid, known for their powerful contractions. Further, the orientation of the LPM fibers, as described by Miller et al. [62], diverges in the medio-lateral and supero-inferior directions relative to the MC. This unique fiber arrangement endows the LPM with a capacity for a diverse range of directional pulls. Such an anatomical feature is essential for the LPM’s role in complex jaw movements, including lateral deviations and protrusions, highlighting its functional versatility within the masticatory system. The understanding of this alignment not only provides insight into the LPM’s functional capabilities but also underlines its distinctiveness from more power-oriented muscles in the jaw apparatus.

The classification of the LPM as pennate or non-pennate is a nuanced subject, with research revealing a spectrum of structural complexities. Murray et al. suggested that the internal architecture of the LPM, including the inner tendon lamellae in the ILPM and non-parallel bundles in the SLPM, hints at a pennate structure [63]. This architecture, coupled with the muscle’s complex innervation pattern, seems to support the existence of functionally heterogeneous zones within the LPM, potentially allowing for a range of force vectors on the condyle.

In contrast, criticizing Murray’s work, McMillan argued that the evidence for a pennate structure within the LPM is not compelling [64]. Although the muscle exhibits internal connective tissue and non-parallel fiber groups, the relatively longer fibers and sarcomeres, uniform fiber length, and minimal tendinous tissue suggest only limited pennation, especially when compared to other jaw muscles like the masseter, temporalis, and medial pterygoid [64].

Further complicating the picture, Foucart et al. reported that the LPM consistently shows multipennate organization, characterized by several muscle layers divided by horizontal aponeuroses [55]. Similarly, Widmalm et al. [22] identified a complex multipennate structure in the origin of the ILPM. Van Eijden’s findings added a subtle variation to this understanding, noting slight pennation in the ILPM in one cadaver [65].

El Haddioui et al. contributed a compelling perspective, suggesting that the LPM exhibits a typical penniform structure similar to that of the masseter and medial pterygoid muscles [5]. This study, conducted in tandem with analyses of other mandibular elevators, posits that despite the distinct insertions of its fibers at each end, the LPM functions as a single unit, primarily due to its penniform structure. El Haddioui et al. (2005) found by plane-by-plane dissections that the LPM comprises eight alternating musculo-aponeurotic layers arranged horizontally and making up a typical penniform structure [5]. These authors referred their results to previous studies of Gaudy et al. [66] and Bravetti et al. [67] to confirm the penniform structure of the LPM through multiple studies. It was suggested that the articular tubercle of the temporal squama serves as a reflexion pulley for the superior musculo-aponeurotic layers [5,12]. However, the publication by El Haddioui et al. (2005) includes a coronal MRI scan in Figure 35 in the ref. [5], where the masticatory muscles were misidentified: the LPM was indicated as the masseter, the masseter was indicated as the medial pterygoid, the medial pterygoid was indicated as the mandible, while the last label in the figure was not explained in the respective caption.

In summary, the LPM’s structural classification is not straightforward. While some studies support multipennate organization, others suggest only limited or no pennation, highlighting the diversity in the structural arrangement. This variance could be attributed to individual anatomical differences, methodological approaches in studies, or a genuine heterogeneity in the muscle’s architecture. The debate over the pennate versus non-pennate classification underscores the complexity of the LPM and the necessity for further, more detailed anatomical research. The physiological characteristics of masticatory muscles depend not only on each masticatory pattern. Still, they could be influenced by the vectorial force resulting from the combined pennation pattern of various bundles [68]. Pennation of a masticatory muscle exists down to the ultrastructural level, where pennate sarcomeres could be distinguished [68]. Unfortunately, the ultrastructure of the LPM was poorly investigated.

### 4.1. Structural Variants of the Lateral Pterygoid Muscle Insertion

**Figure 5:**
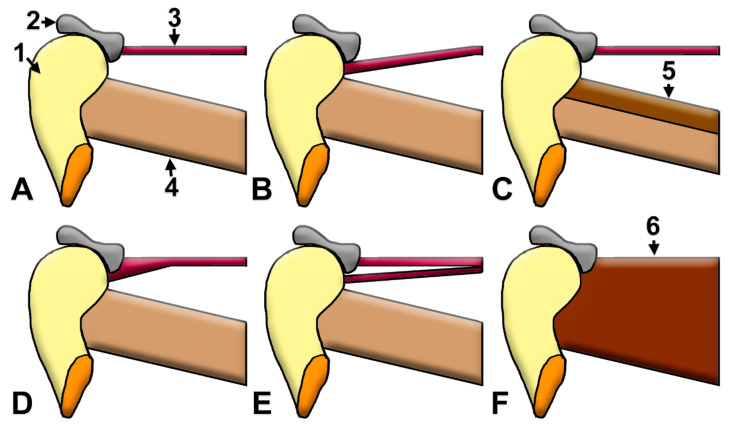
Diagrams of different lateral pterygoid muscle (LPM) insertion possibilities and number of heads: 1. mandibular condyle (MC); 2. temporomandibular joint disc (TMJD); 3. superior head of the lateral pterygoid muscle (SLPM); 4. inferior head of the lateral pterygoid muscle (ILPM); 5. middle head of the lateral pterygoid muscle (MLPM); 6. unsplit LPM. Moreover, **A**: the SLPM inserts on the TMJD and the ILPM on the MC; **B**: both the SLPM and ILPM insert on the MC; **C**: the SLPM inserts on the TMJD and the MLPM and ILPM insert on the MC; **D**: the unsplit SLPM inserts on the TMJD and MC and the ILPM on the MC; **E**: a split SLPM inserts on the TMJD and MC and the ILPM on the MC; and **F**: unsplit LPM.

The anatomical insertion sites of the SLPM and ILPM demonstrate significant variability (Figure 5), which has led to the proposal of multiple classification systems [60]. In 55% of cases, an asymmetric right–left LPM organization was found [55]. Patterning only the SLPM [18,29,37] could not result in an accurate patterning of the LPM, as the ILPM could have a discal bundle [28,35]; thus, it could not be assumed in all cases to be a mandibular-only insertion. These latter range from two to four types and reflect the observed insertion patterns. For example, Type 1 is characterized by various insertion modalities for the SLPM, including the TMJD alone, the TMJD in conjunction with the MC, or as two distinct bundles, differing between authors [60]. Type 2 mirrors Type 1 regarding the SLPM’s insertions but differs from it by the SLPM insertion on the TMJD and MC as a single bundle [60]. Type 3 is defined by the SLPM potentially inserting as a single bundle on the TMJD or the MC, and the ILPM on the MC, and also includes the possibility of an MLPM attaching to the MC [60]. In Type 4, the SLPM inserts on the TMJD and the ILPM inserts on the TMJD and MC [60]; therefore, the ILPM insertion on the MC may not be exclusive, as illustrated in Table 1. However, most of the SLPM inserts into the MC, and the contraction of the little part of the SLPM, which inserts to the TMJD, should not result in an anterior disc displacement [29]. The pattern of SLPM insertion into the TMJD does not have a predictive or prognostic value for TMJ internal derangement [18]. Conversely, there is a link between TMJD displacement and LPM pathologic changes [42].

The attachment of the SLPM to the TMJD is widely recognized [18,35,37,39,41,42]. Naidoo (1996) found in a dissection study that in 65% of cases, the SLPM attached to the medial aspect of the TMJ capsule, TMJD and MC; in 27.5%, it was not attached to the TMJD; and in 7.5% of cases, peculiar possibilities for the LPM insertion on the TMJD were found: SLPM wholly attached to the TMJD, and a bundle of the ILPM inserted into the TMJD [28]. However, the findings of Christo et al. [31], in just ten dissected TMJs, dispute this because they did not find the TMJD insertion of the SLPM. In cases where the SLPM was not inserted into the TMJD, Naidoo (1996) found that the epimysium of the SLPM was continuous with the anterior attachment of the TMJD to the articular tubercle [28].

### 4.2. Variable Number of Heads and Bundles of the Lateral Pterygoid Muscle

Based on morphological studies and innervation studies, the LPM is considered to have no clear divisions [32]. Bertilsson and Ström (1995), consistently quoted in Bergman’s *Comprehensive Encyclopedia of Human Anatomic Variation* [69], documented different studies and observed that the LPM was equally described as consisting of two heads or without separate parts, therefore being single-headed [70]. When the two authors documented several publications indicating that the LPM is organized into three parts, the additional one they recorded as a medial head of the LPM inserted into the extreme medial portion of the articular capsule [70]. Still, a clear origin site for this third head was not indicated.

**Figure 6:**
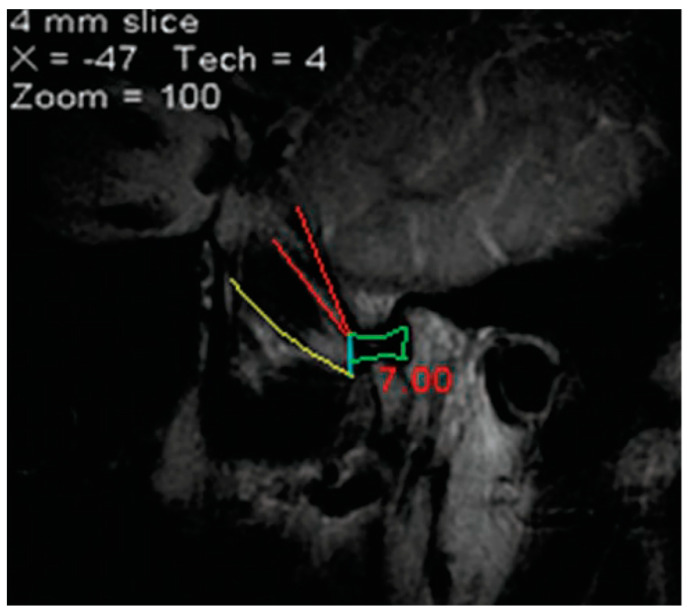
In the caption of Figure 1 in Pompei Filho’s paper [74], which was published under a CC BY-NC 4.0 license, the authors indicated: “MRI image of the TMJ, the superior and inferior margins of the third head of the lateral pterygoid muscle are bound with red. In yellow is observed in the inferior margin of the THLPM, with its insertion area into the disc in blue. The joint disc narrows in green.” THLPM is the abbreviation the authors used for the third head of the lateral pterygoid muscle [74]. The authors drew red lines on the margins of that THLPM, then they drew a lower yellow line to indicate, again, the inferior margin of the THLPM. The insertion area on the TMJ disc (blue line) is larger than the marked green area of the disc. We could not understand the meaning of the sentence “The joint disc narrows in green”. It was used as a measurement that is not explained in the caption of that figure. The osseous landmarks are not indicated and that MRI image was not oriented anatomically.

Abe et al. [71] found a single-headed LPM in 53% of subjects, contrasting with Bertilsson et al.’s [70] observation of a 27% occurrence. Gaudy (1993), analyzing 300 subjects, proposed the LPM as predominantly a single-headed muscle [66]. The variation in the maxillary artery’s position might influence this discrepancy, where a superficial artery correlates with a more homogenous LPM structure, limiting the separation of the pterygoid insertions [55]. A three-headed LPM has been found by numerous authors [4,21,37,41,55,71,72,73]. Indicating the third head to be a medial one further complicates attempts at anatomically patterning the LPM. Pompei Filho et al. (2009) estimated the prevalence of a third head of the LPM at 20.22%, with its insertion being entirely on the TMJ disc [74]. The authors speculated the role of this third head of the LPM in the development of TMJ disc function alterations and anterior disc displacements [74]. For their publication, Pompei Filho et al. (2009) used unique MRI evidence that was interpreted in a contradictory manner (Figure 6). Therefore, caution should be taken when different publications are included in reviews, such as in that of Stockle et al. (2019) [4]. One should note here the observation of Birou et al. (1991), who performed anatomic sections and CT determinations in 39 specimens and observed that as many as three parts of the LPM can be distinguished, with the head classically called pterygoid being itself divided in two by a septum [75]. The authors discussed that this cannot be attributed to an artefact of dissection since the CT sections were made before the anatomic sections and certainly related to a reinforcing fascia and not to a supplementary muscular head [75]. Misjudging the intramuscular septa may indeed lead to an increasing number of the heads of the LPM.

#### 4.2.1. The Single-Headed Lateral Pterygoid Muscle

**Figure 7:**
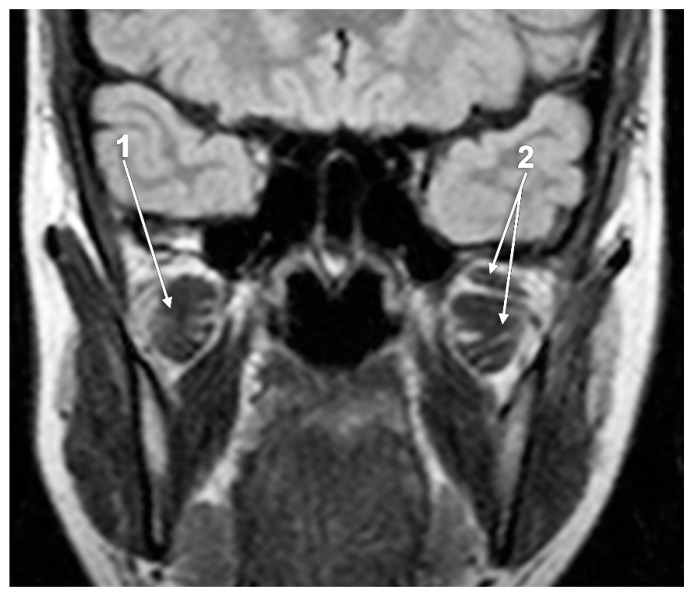
Coronal MRI slice. Bilateral morphological asymmetry of the lateral pterygoid muscles, single-headed (1) and double-headed (2).

Researchers have identified single-headed LPMs [71,72,73,76]. In 25 dissected LPMs, a single-headed LPM was found in 15% [72]. This results when the SLPM misses (Figure 7).

#### 4.2.2. The Lateral and Medial Bundles of the Superior Head of the Lateral Pterygoid Muscle

**Figure 8:**
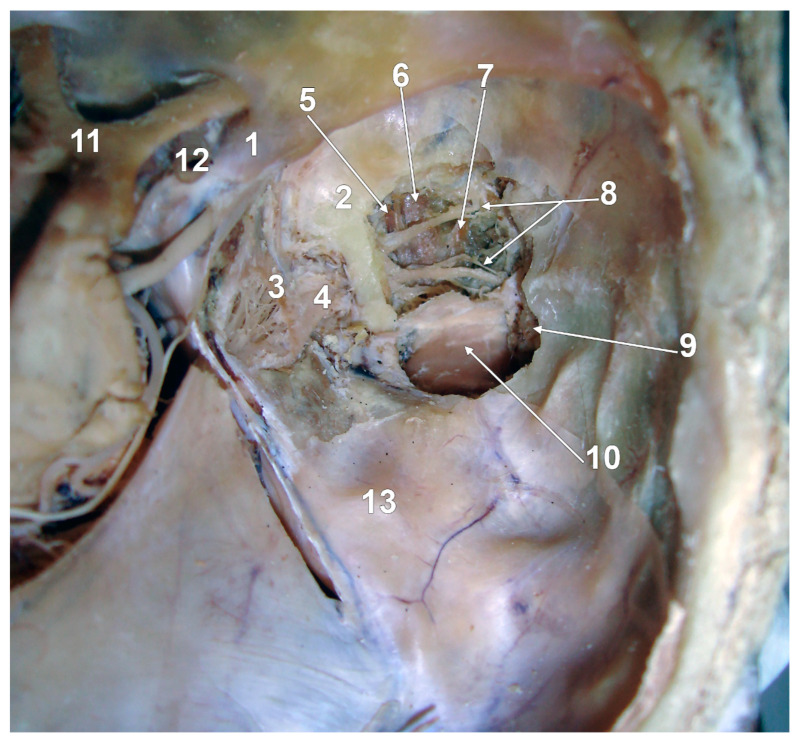
The superior head of the lateral pterygoid muscle (SLPM) consists of three macroscopically distinctive bundles. Superior approach of dissection, via the floor of the middle cranial fossa. Right side: 1. anterior clinoid process; 2. greater sphenoidal wing; 3. trigeminal ganglion; 4. mandibular nerve entering the foramen ovale; 5. medial bundle of the SLPM; 6. intermediate bundle of the SLPM; 7. lateral bundle of the SLPM; 8. deep temporal nerves; 9. temporal m.; 10. TMJ disc; 11. optic chiasm; 12. internal carotid a.; and 13. arcuate eminence of the petrous bone.

A dissection study found, in 20% of cases, three heads of the LPM: superior, inferior, and medial [72]. A recent systematic review of the LPM used a hemisected head to present an SLPM consisting of a medial and a lateral bundle [4]. We used that superior approach of the SLPM and observed distinctive bundles of it, medial, intermediate and lateral (Figure 8). Sugisaki et al. (1986) dissected 26 TMJs by the superior approach and found that the LPM varied with the cadaver in the number of heads, which indicates that the muscle is substantially different from cadaver to cadaver [21]. They did not find the variant we present here in Figure 8. However, a single case may not be regarded as a common pattern. Nevertheless, as Abe et al. (1997) discussed, since the SLPM and ILPM approach the insertion combined and entangled, it is difficult to determine where the muscle bundles originate even when using the superior approach [26]. The possibility of error exists when determining the LPM attachment through the superior approach method or on slices [26]. It is only possible to make a correct determination by tracing each individual muscle bundle under a microscope [26].

**Figure 9:**
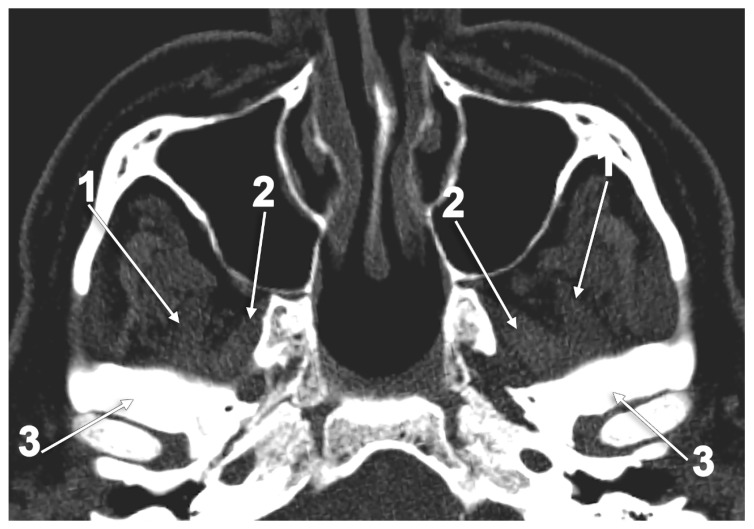
Axial slice through the superior heads of the lateral pterygoid muscles (SLPMs): 1. lateral bundle of the SLPM; 2. medial bundle of the SLPM; and 3. articular tubercle.

Fujita et al. (2001) found a third inner head of the LPM that originated on the surface of the greater wing of the sphenoid bone [30], just as a typical SLPM does. The prevalence of such a medial head of the LPM, as a result of MRI scans, is 20.22% [74]. Sagittally divided SLPMs can be distinguished on axial CT slices (Figure 9) and dissections [30].

#### 4.2.3. The Lateral and Medial Bundles of the Inferior Head of the Lateral Pterygoid Muscle

**Figure 10:**
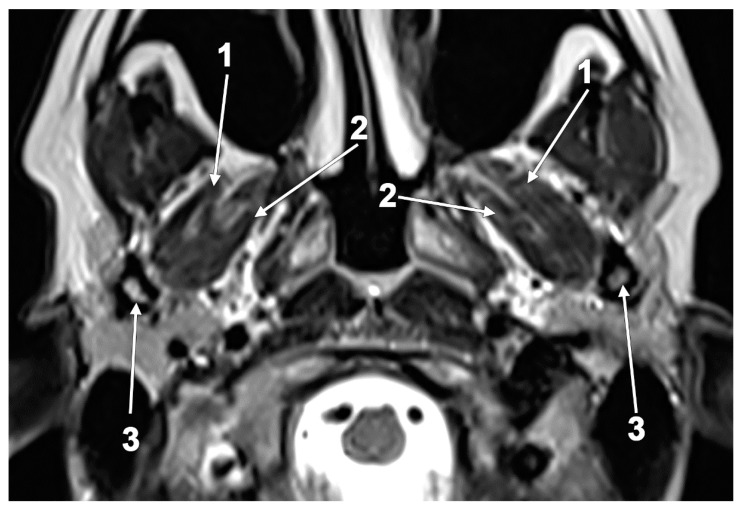
Axial MRI slice through the inferior heads of the lateral pterygoid muscle (ILPMs). Inferior view: 1. lateral bundle of the ILPM; 2. medial bundle of the ILPM; and 3. neck of the mandible.

A dissection study of just five human cadaver heads sought to classify the possibilities of insertion of the SLPM [23]. The authors observed an extra medial head of the LPM in one joint, resulting in a sagittal split of the ILPM [23]. The medial and lateral bundles of the ILPM could be easily observed on axial slices (Figure 10).

**Figure 11:**
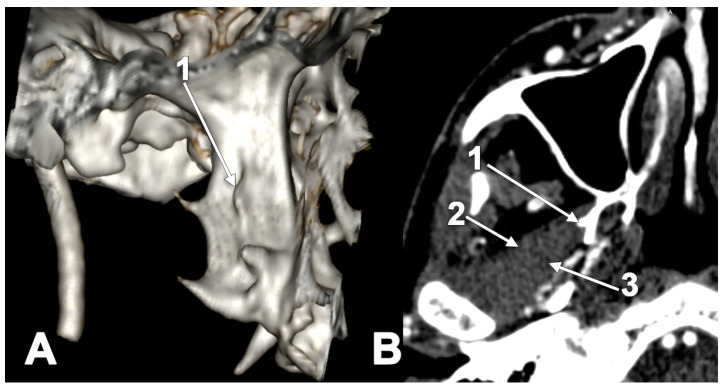
Outer vertical ridge of the lateral pterygoid plate that separates the origins of the medial and lateral bundles of the inferior head of the lateral pterygoid muscle (ILPM). **A**. Three-dimensional rendering of the right lateral pterygoid plate, lateral view. **B**. Axial slice, viewed inferiorly: 1. vertical ridge of the lateral pterygoid plate; 2. lateral bundle of the ILPM; and 3. medial bundle of the ILPM.

A vertical ridge could separate the origins on the lateral pterygoid plate: the lateral bundle of the ILPM attaches anteriorly to that ridge and the medial one posterior to it (Figure 11). They can merge posteriorly or insert distinctively on the external and internal parts of the pterygoid fovea.

#### 4.2.4. Nerves, Arteries, and the Lateral Pterygoid Muscle

In 52 dissected sides, different nerves were found passing through the medial fibers of the inferior head of the LPM: the auriculotemporal, inferior alveolar, lingual, anterior deep temporal, and mylohyoid nerve [77].

**Figure 12:**
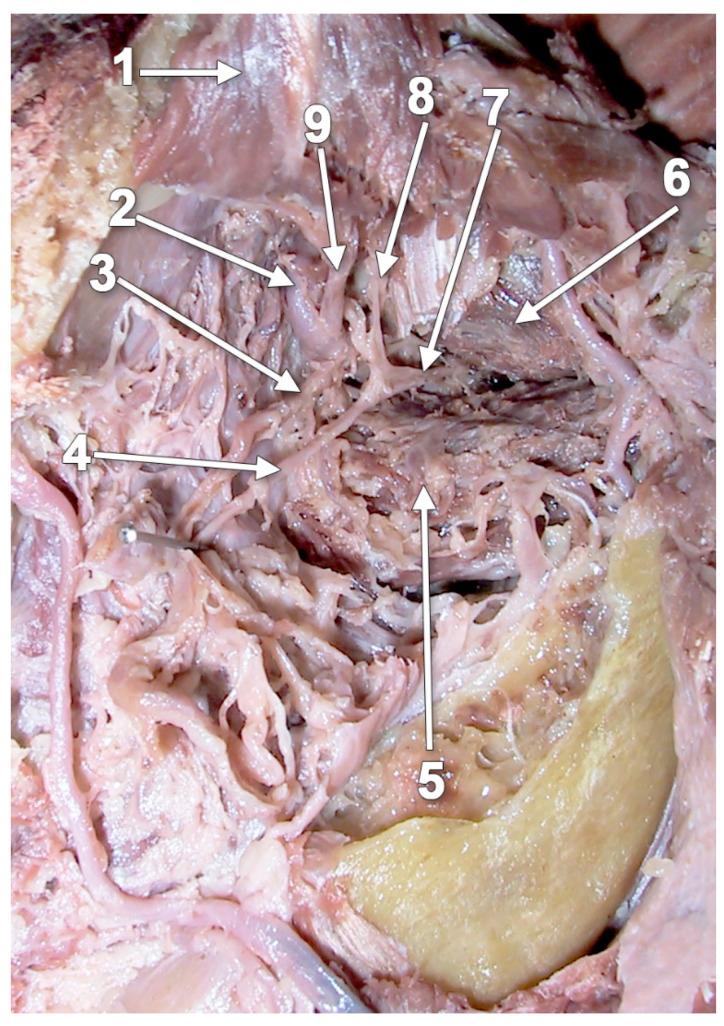
Dissection of the left lateral pterygoid muscle. The upper part of the mandibular ramus was removed. Lateral view: 1. temporal m. (reflected superiorly); 2. maxillary artery (deep variant); 3. buccal artery; 4. buccal nerve; 5. inferior head of the lateral pterygoid muscle; 6. superior head of the lateral pterygoid muscle; 7. temporobuccal trunk coursing between the two heads of the lateral pterygoid muscle; 8. anterior deep temporal nerve; and 9. anterior deep temporal artery.

The SLPM and ILPM are typically separated by a groove in the anterior part of the LPM [32]. By dissection, Usui et al. (2008) found that the buccal nerve formed a common trunk with the anterior deep temporal nerve, mainly passing between the SLPM and ILPM [32]. The courses of that temporobuccal common trunk (TBT) were classified into three types: type 1, the TBT ran on the superior surface of the muscle (3.4%); type 2, the TBT ran through the groove between the SLPM and ILPM (74.5%); and type 3, the TBT coursed through the ILPM (22.3%) [32]. Type 2 is sampled in Figure 12.

The maxillary artery courses either lateral/superficial or medial/deep in relation to the LPM; an intermediate type is represented by a course of the maxillary artery through the LPM [78]. When coursing deep into the ILPM, the maxillary artery emerges anteriorly between the ILPM and SLPM (Figure 12). The variable course of the maxillary artery in relation to the LPM was studied previously [79,80,81,82]. Seemingly, the maxillary artery is more likely to pass deep into the ILPM in females than in males [83]. Therefore, the LPM and the maxillary artery are key anatomical landmarks for the ultrasound-guided trigeminal nerve block during which the needle passes through the LPM [84].

## 5. Modified Origin Sites of the Lateral Pterygoid Muscle

### 5.1. Broad Lateral Pterygoid Plates

**Figure 13:**
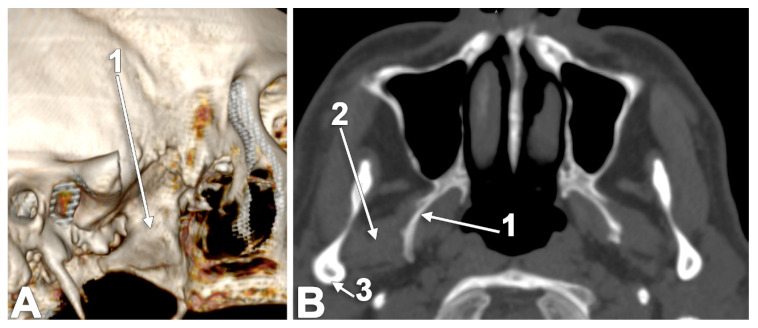
Bilateral broad lateral pterygoid plates serve as the origins of the inferior heads of the lateral pterygoid muscles. (**A**) Three-dimensional volume rendering. Right side. Lateral view. 1. lateral pterygoid plate (**B**) Axial slice through the pterygoid plates: 1. broad right lateral pterygoid plate; 2. inferior head of the lateral pterygoid muscle; and 3. neck of the mandible.

Sometimes, the pterygospinous ligament is not ossified, but the lateral pterygoid plate extends to the sphenoidal spine [85]. Such broad lateral pterygoid plates expand the surface of the origin of the ILPMs (Figure 13).

### 5.2. The Pterygospinous and Pterygoalar Bars

**Figure 14:**
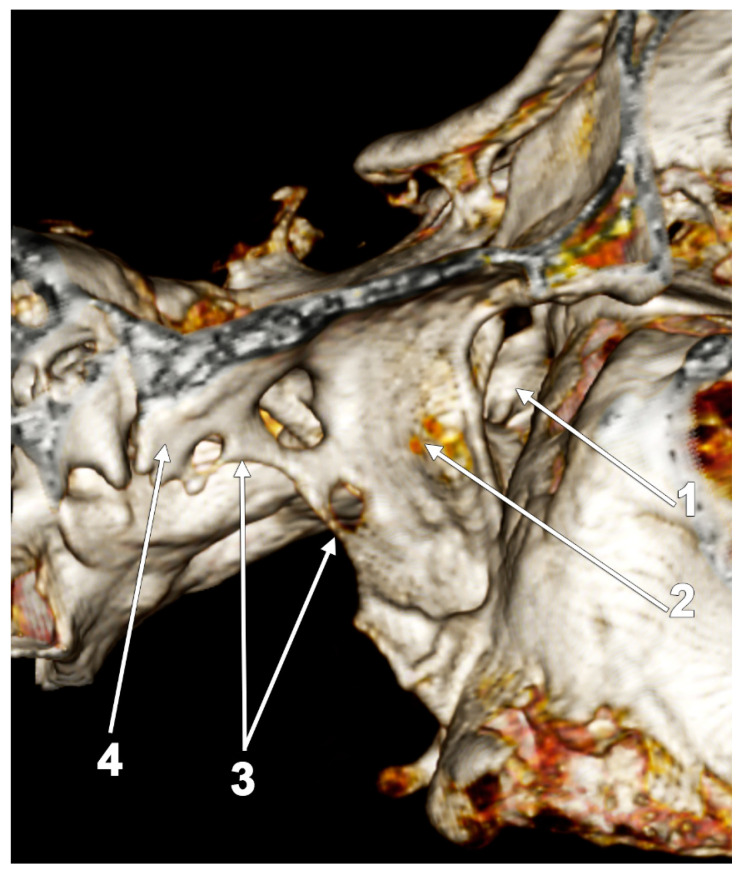
Three-dimensional volume rendering of the right pterygoid process. Infero-lateral view. Complete pterygospinous bar: 1. pterygopalatine fossa; 2. lateral pterygoid plate; 3. pterygospinous bar; and 4. sphenoidal spine.

The pterygospinous ligament of Civinini extends from the posterior margin of the lateral pterygoid plate to the sphenoidal spine of the greater wing [86]. When it ossifies, it results in Civinini’s pterygospinous bar and, above it, the pterygospinous foramen [86]. The pterygoalar ligament extends from the lateral pterygoid lamina to the greater sphenoidal wing; it may ossify, resulting in the pterygoalar bar [86]. A complete pterygospinous bridging was found bilaterally in 1/50 skulls [86]. Such morphology is presented in Figure 14.

**Figure 15:**
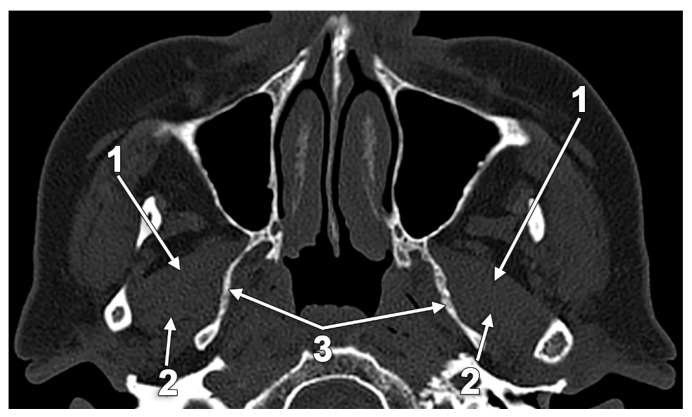
Axial CT slice through bilateral pterygospinous bars that attach fibers of the lateral pterygoid muscles’ inferior fascicles (ILPMs): 1. lateral fibers of the ILPM; 2. medial fibers of the ILPM; and 3. pterygospinous bars of Civinini.

The pterygospinous and pterygoalar bars may have different morphologies [87]. They increase the surface available for the origin of the ILPM (Figure 15).

## 6. Aberrant Insertion Sites and Accessory Bundles of the Lateral Pterygoid Muscle

As each of the TMJ’s components progressively develops from a common mass of embryonic mesenchyme interposed between the future temporal bone and mandibular regions [88,89], anatomical variations of the LPM should be expected. The variations among the morphological studies of the LPM may well be explained by fundamental differences in the ages of the study samples—that is, embryos, fetuses, and adults [89].

### 6.1. The Extraincisural Insertion of the Lateral Pterygoid Muscle

Various transitional muscle bundles have often been observed in the lateral part of the masticatory muscles [34]. The outer fibers of the ILPM could diverge postero-laterally and pass through the mandibular notch to an extraincisural insertion on an accessory pterygoid fovea of the MC [60].

#### The Accessory Pterygoid Fovea

An accessory pterygoid fovea was found unilaterally on the lateral condylar tubercle outside the notch of the mandible [60]. Therefore, the LPM was inserted on the entire width of the MC, including the accessory pterygoid fovea [60].

### 6.2. The Pterygoideus Proprius Muscle

**Figure 16:**
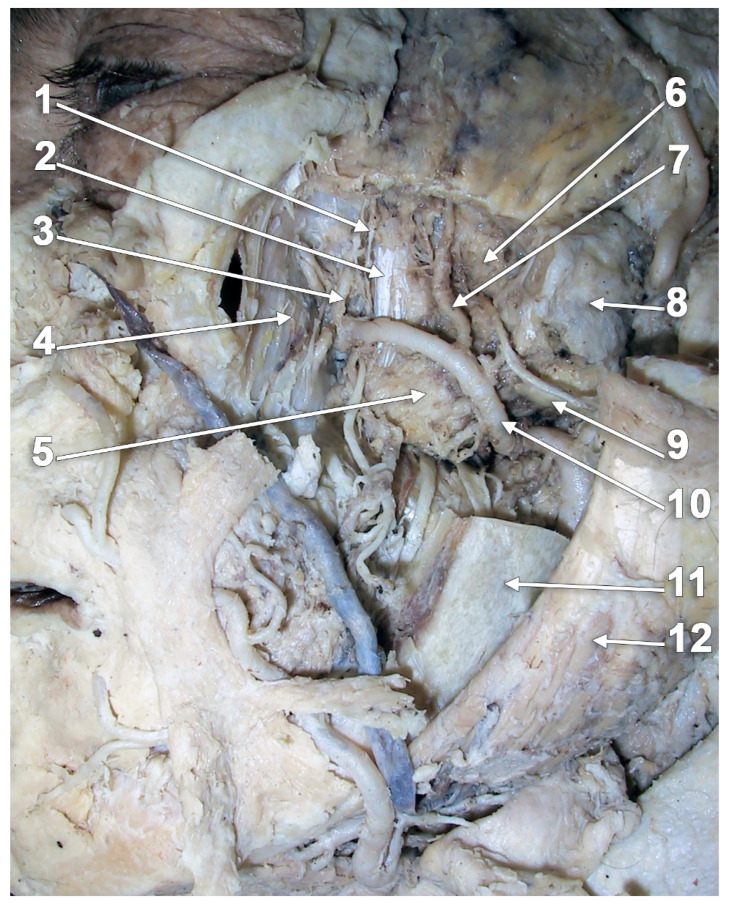
Dissection of the left infratemporal fossa. The pterygoideus proprius muscle was found: 1. deep anterior temporal nerve; 2. pterygoideus proprius muscle; 3. deep anterior temporal artery; 4. maxillary tuberosity; 5. inferior head of the lateral pterygoid muscle; 6. superior head of the lateral pterygoid muscle; 7. deep posterior temporal artery; 8. temporomandibular joint; 9. neck of the mandible; 10. maxillary artery; 11. ramus of the mandible; and 12. masseter muscle.

The pterygoideus proprius muscle (PPM) was discovered by Henle in 1858 [90]. Different authors further reported Henle’s muscle [91]. The PPM was found in 1.26% of the cadaveric head halves in dissection studies, but in in vivo studies using magnetic resonance, it occurred in 12.82% of head halves [91]. The PPM arises from the infratemporal crest of the greater sphenoidal wing and inserts inferiorly into the posterior border of the lateral pterygoid plate; it may also reach the maxillary tuberosity, the pyramidal process of the palatine bone, or the pterygomandibular raphe [47]. It may also connect with the medial surface of the temporal muscle [47]. The PPM is rarely well developed [47]. When a PPM is present, the TBT emerges in front of it to divide into the anterior deep temporal and buccal nerves (Figure 16), which could supply it with nerve fibers. An arterial supply of the PPM from the deep temporal arteries can be observed in Figure 15. The PPM has variable attachments on the masticatory muscles, bones, and ligaments; thus, it can create different force directions during contraction and alter the normal TMJ mechanics [91].

### 6.3. The Maxillomandibularis Muscle

Fibers of the ILPM occasionally originate on the posterior surface of the maxilla to configure a distinctive maxillomandibular muscle adjacent to, but not merged with, the ILPM [92].

### 6.4. The Pterygospinous Muscle

**Figure 17:**
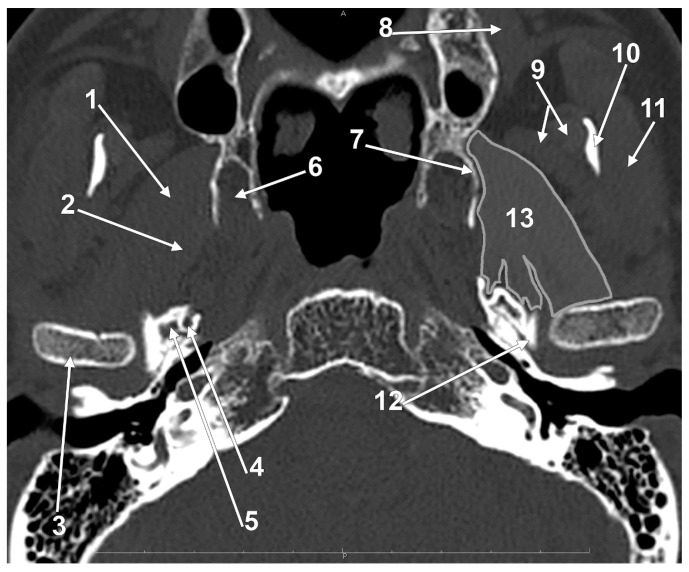
Axial CT slice through the upper parts of the pterygoid plates. Inferior view. Pterygospinous bundles of the lateral pterygoid muscles: 1. inferior head of the right lateral pterygoid muscle; 2. pterygospinous muscle; 3. head of the mandible; 4. spinous foramen; 5. sphenoidal spine; 6. medial pterygoid muscle; 7. lateral pterygoid plate; 8. buccinator muscle; 9. temporal muscle; 10. coronoid process; 11. masseter muscle; 12. endoglenoid process; and 13. inferior head of the left lateral pterygoid muscle.

A pterygospinous muscle (Figure 17) that is either lateral to the pterygospinous ligament or replaces it is apposed on the lateral surface of the medial pterygoid muscle [47]. Lang (1995) quoted Kreutzer (1896) and Macalister and Thanc (1898), who indicated that the pterygospinous muscle can be identified in about 50% of specimens.

### 6.5. The Pterygofacialis Muscle

Lang (1995) quoted Kreutzer (1896) and Grüber (1872) and described the pterygofacialis muscle as arising from the pterygospinous ligament or an accessory medial or lateral ligament [47]. Such a pterygofacialis muscle may be inserted either on the medial pterygoid muscle, on the posterior margin of the mandibular ramus at the level of the mandibular foramen, or on the pterygoid fossa [47].

### 6.6. The Sphenomandibular Muscle

Dunn et al. (1996) provided the first evidence of “a hitherto unreported, functionally distinct craniomandibular muscle as observed in 25 cadaveric specimens and MRI scans of clinical patients”, which was named “sphenomandibularis” [93]. The sphenomandibular muscle (SMM) was consistently observed to originate as a distinct muscular structure from the skull’s base, not the skull’s lateral surface, the commonly described location of origin for the temporal muscle [94]. The insertion of the SMM was also distinct from that of the temporalis [93]. The SMM is more similar to the LPM than the temporal muscle [94]. The SMM has an innervation pattern more similar to the LPM than the temporalis and exhibits different electromyographic patterns, other than the temporal muscle, during various stages of the chewing cycle [94,95].

## 7. The Volume of the Lateral Pterygoid Muscle

The volumes of the masticatory muscles determine the individual topographic anatomy of the clinically relevant masticatory spaces and may be age-dependent [96]. Being directly connected to the TMJ, the LPM is one of the most important muscles in mastication physiology [97].

The size (cross-sectional area, length, and volume) of the human jaw muscles varies with the craniofacial form [98]. Goto et al. (2006) studied the size and orientation of these muscles in patients with mandibular laterognathism and compared the results in the patient group with those in the control group [98]. Regarding the muscle orientation on the deviated side compared with the opposite side, no difference was found in the patient group for the LPM [98]. Both sides of the LPM in the patients showed a more medially directed axial angle compared with those in the controls [98]. The LPM showed only a smaller volume in the patient group [98]. The inter-individual variations in the muscle shape were larger in the patients than in the controls [98].

A different study found differences according to the TMJ disc position: the volume and area of the LPM were significantly different between closed and open mouth positions [97]. Significant differences in the volume and area of the masticatory muscles were found between females and males [97].

Weijs and Hillen (1984, 1985) pointed to a blurred zone regarding the masticatory muscles: their forces of action [99,100]. They correctly underlined that the force of a muscle is not only determined by physiological parameters but also by anatomical ones, such as the physiological cross-section (PCS), i.e., the total cross-section of all the muscle fibers [99]. They also observed that most studies at that time used the PCS of the masticatory muscles given by Schumacher in 1961 [61,100]. They raised doubts as to Schumacher’s data because they were derived from cadavers with a high mean age that lacked most natural teeth and probably had atrophied muscles, and they could not be of use in conjunction with experimental data from young individuals with complete dentitions [99,100]. Moreover, using mean cross-sections does not take into account the individual variations, such as the skull variations [100]. We listed here some additional variables for the LPM: the variable structure, the attachment sites and the accessory muscular bundles. Therefore, little is known about the size of the jaw muscles and their individual geometry could not be predicted. This means that neither the PCS nor force of the LPM could be anticipated and so should be evaluated on a case-by-case basis. The study by Weijs and Hillen in 1985 supports this, because they found strong correlations in the cross-sectional area only for masseter and medial pterygoid muscles [99]. The PCSs in their study were significantly larger than the ones found in cadavers by Schumacher (1961) [61,99].

Goto et al. (2002) found that the LPM significantly decreases its volume during jaw opening [101]. The authors discussed that the physiological reason for the decrease in the volume of the LPM after jaw opening remains unclear [101]. They listed several possible explanations: (1) a decrease in the lengths of the muscle fibers when the muscle contracts; (2) compression of the LPM by the condyle; and (3) changes that may occur in the regional blood flow [101]. Although they used MRI scans for that study, these authors considered just a two-headed model of the LPM in which the change in volume of the LPM may reflect the shortening of the ILPM [101].

The volume of the LPM was assessed in unilateral chewing and it was observed that the volumes of both the ILPM and SLPM at the affected side were significantly greater than those of the unaffected side [44]. The authors demonstrated that systematically sampled MRI slices through the LPM can be used to obtain unbiased estimates [44].

Computed tomography was used to evaluate, in 60 individuals, the relationship between mandibular prognathism and the LPM size [102]. In the prognathic group were found smaller LPM volume/length ratios compared to the normal group [102]. In addition, the normal group displayed a larger horizontal angle to the mandibular and palatal planes than the prognathic group [102]. These results were thought to support the association between the mechanical drawback of the LPM in the prognathic group and the mandibular prognathism [102]. The respective findings were supported by a list of just 19 references [102], which may signify a certain degree of novelty.

The morphological and functional changes in the LPM caused by jaw movements could be sensitively detected by diffusion tensor imaging in vivo, which may serve as a non-invasive method for simultaneously investigating the functional and morphological features of the LPM during jaw movements [40].

### The Atrophy and Hypertrophy of the Lateral Pterygoid Muscle

The concept of atrophy in the LPM is complex, with various studies presenting different definitions and observations. Kiliç et al. [35] noted muscular atrophy in several specimens without explicitly defining what constitutes atrophy. Conversely, Imanimoghaddam et al. [42] described atrophy as areas of high signal on proton-density and T2 images, indicative of fat tissue substitution in the upper lobe, and observed a 21.3% prevalence, a higher percentage compared to the prevalence obtained by D’Ippolito et al. of 2.85% [103].

Yang et al. (2002) [104] characterized LPM atrophy as a fatty replacement marked by high signal areas on MRI images, identifying hypertrophy, atrophy, and/or contracture in either or both heads of the LPM. Similarly, Yang et al. (2001) [105] defined LPM atrophy as high signal areas on MRI, combined with fatty replacement and either no change or a reduction in muscle size, particularly in the SLPM but not in the ILPM.

Schellhas et al. [106] connected diffuse masticatory muscle atrophy to systemic illnesses or mass lesions affecting the trigeminal ganglion. They observed that atrophic muscles often show increased MRI signals due to dystrophic changes and fatty replacement. They also noted the challenge in distinguishing between muscle hypoplasia and acquired atrophy, especially with unusual glenoid fossa configurations.

Finden et al. [107] associated increased T2 signal intensity in the LPM with pathological alterations in the condylar head and TMJD relationship, suggesting that this intensity might indicate muscular edema or fatty changes due to atrophy.

Furthermore, Taskaya-Yilmaz et al. [19] found a higher incidence of atrophy in TMJs with anterior disc displacement without reduction. Predominantly, atrophy was seen in the superior head of the LPM, with the fatty replacement on MR images used as a visual indicator of muscle atrophy. They also noted a higher occurrence of atrophy in type 1 muscle attachment compared to type 2, implying a potential link between muscle attachment type and atrophy.

The hypertrophy in the LPM is detailed in various studies, each offering insights into its prevalence, defining characteristics, and potential causes. Imanimoghaddam et al. [42] reported a 5% prevalence of hypertrophy in the LPM. They described hypertrophy as an increase in the mid-portion muscle size of each lobe of the SLPM, characterized by bulging of both the upper and lower edges. Lopes et al. (2015) observed that patients with temporomandibular disorders and migraine tend to have hypertrophy of the LPM (58.7%) [108].

Yang et al. (2001) [105] found that abnormalities in both bellies of the LPM often involved hypertrophy of the ILPM, alongside other changes in the SLPM. They observed that hypertrophy was the most common finding in the SLPM (54.9%) and the only finding in the ILPM. Furthermore, they noted that pathological changes in the SLPM were more frequent than in the ILPM, with hypertrophy of the ILPM often appearing in combination with various pathological changes in the SLPM. This was interpreted as possibly indicative of the SLPM being abnormally overloaded to maintain stability.

In their 2002 study, Yang et al. [104] described hypertrophy of the LPM as an apparent enlargement in the middle part of the muscle belly, with the upper and lower edges forming convex curves. They attributed this hypertrophy to excessive work and overloading of the skeletal muscles.

Grunert et al. [109] observed cases of bilateral SLPM hypertrophy and posited that such hypertrophic changes might result from chronic overload.

D’Ippolito et al. [103] noted a lower prevalence of hypertrophy at 1.45%, defining it as a muscle enlargement with a homogeneous signal in MRI sequences. This enlargement was visually identified as a convex curve in the medial portion of the upper or lower muscle border in sagittal section sequences.

Together, these studies present a comprehensive view of LPM hypertrophy, highlighting its varied prevalence and potential link to overuse and overload of the muscle. Understanding hypertrophy in the LPM is crucial for diagnosing and managing conditions associated with the masticatory apparatus.

## 8. Conclusions

The vectorial force of the normal LPM is modified by the highly variable internal structure and general morphology. The atypical origin and insertion sites, as well as the rare accessory muscles (pterygoideus proprius, pterygotemporal, maxillomandibularis, pterygospinous, pterygofacialis, and sphenomandibular), increase the diversity of the LPM.

The general anatomical theory of the LPM is seemingly limited to the following: it is a structurally and functionally heterogeneous masticatory muscle that acts upon the mandibular condyle and, in most cases, the TMJ disc. Being anatomically unpredictable, a personalized evaluation of the LPM should be considered using modern imaging methods, if available.

The previously reported evidence resulted from diverse methodological approaches. The conclusions that were reached do not fit together to support a general anatomical theory.

More studies that may convincingly use MRI scans on large numbers of patients are urged to support a pertinent theory on the LPM’s structure. However, further research should concomitantly evaluate the osseous support, as this could also vary and contribute to functional deviations from the typical function.

## Figures and Tables

**Table 1:** Different anatomical types of the lateral pterygoid muscle (LPM) and prevalence. The anatomical descriptions of the superior (SLPM) and inferior (ILPM) heads of the LPM are author-specific [19,21,22,23,24,56,57]. A middle head (MLPM) of the LPM was recorded distinctively. TMJD: temporomandibular joint disc; MC: mandibular condyle.

First Author, Year	TYPE I		TYPE II		TYPE III		TYPE IV	
	Type	%	Type	%	Type	%	Type	%
Kiliç, 2010 [35]	SLPM > TMJD, MC ILPM > MC	36.7	SLPM > TMJD ILPM > MC	28.6%	SLPM > MC ILPM > MC	26.5%	SLPM > TMJD ILPM > TMJD, MC	8.2%
Dergin, 2012 [39]	SLPM > TMJD ILPM > MC	29.6	SLPM > TMJD, MC ILPM > MC	40.8	SLPM > TMJD ILPM&MLPM > MC	29.6	-	-
Litko, 2016 [41]	SLPM > TMJD ILPM > MC	7.6	SLPM > TMJD, MC ILPM > MC	66.7	SLPM > TMJD ILPM&MLPM > MC	25.7		
Imanimoghaddam, 2013 [42]	SLPM > TMJD, MC (2 bundles) ILPM > MC	63.75	SLPM > TMJD, MC (1 bundle) ILPM > MC	23.75	SLPM > TMJD ILPM > MC	12.5		
Taskaya-Yilmaz, 2005 [19]	SLPM > TMJD ILPM > MC	66.9	SLPM > TMJD, MC ILPM > MC	33.1				
